# A Novel Epidemic Model Base on Pulse Charging in Wireless Rechargeable Sensor Networks

**DOI:** 10.3390/e24020302

**Published:** 2022-02-21

**Authors:** Guiyun Liu, Xiaokai Su, Fenghuo Hong, Xiaojing Zhong, Zhongwei Liang, Xilai Wu, Ziyi Huang

**Affiliations:** 1 Guangzhou Industry & Information Technology Institute for Intelligent Robotic Equipment, Guangzhou University, Guangzhou 510006, China; liugy@gzhu.edu.cn; 2 School of Mechanical and Electric Engineering, Guangzhou University, Guangzhou 510006, China; suxkaii@gmail.com (X.S.); hfh15814933585@outlook.com (F.H.); soberlobe@gmail.com (X.W.); hzy1026931135@gmail.com (Z.H.)

**Keywords:** wireless rechargeable sensor network, pulse charging, cyber security, stability analysis, persistence analysis, optimal control

## Abstract

As wireless rechargeable sensor networks (WRSNs) are gradually being widely accepted and recognized, the security issues of WRSNs have also become the focus of research discussion. In the existing WRSNs research, few people introduced the idea of pulse charging. Taking into account the utilization rate of nodes’ energy, this paper proposes a novel pulse infectious disease model (SIALS-P), which is composed of susceptible, infected, anti-malware and low-energy susceptible states under pulse charging, to deal with the security issues of WRSNs. In each periodic pulse point, some parts of low energy states (LS nodes, LI nodes) will be converted into the normal energy states (S nodes, I nodes) to control the number of susceptible nodes and infected nodes. This paper first analyzes the local stability of the SIALS-P model by Floquet theory. Then, a suitable comparison system is given by comparing theorem to analyze the stability of malware-free *T*-period solution and the persistence of malware transmission. Additionally, the optimal control of the proposed model is analyzed. Finally, the comparative simulation analysis regarding the proposed model, the non-charging model and the continuous charging model is given, and the effects of parameters on the basic reproduction number of the three models are shown. Meanwhile, the sensitivity of each parameter and the optimal control theory is further verified.

## 1. Introduction

In recent years, wireless sensor networks (WSNs) have become a hotspot causing extensive attention from researchers [1,2,3,4,5]. Sensor nodes with data storage and data transferring functions make up a sensor network. Nodes can monitor the physical environment near them by deploying in an area without manual monitoring. WSNs have a wide range of applications such as secondary agricultural production, ecological monitoring, traffic safety monitoring, healthcare services and military fields [6]. However, because of the vulnerable nature of nodes and the limited battery capacity, security [7] and short life cycle [8] problems remain to be solved.

Researchers have come up with lots of solutions to optimize energy utilization problems. It is noted that the deployment of rechargeable nodes can solve the energy problem more fundamentally. Wireless rechargeable sensor networks (WRSNs) consist of rechargeable sensor nodes. In recent years, lots of research on WRSNs mainly focuses on the charging planning problem and the energy allocation problem [9,10,11]. However, the network security of WRSNs is rarely studied. Malware can replicate itself. Once implanted into the network system, malware will cause information leakage, network interruption and even network breakdown [12].

Since the infection mechanism of disease in the population is almost the same as the transmission mechanism of malware in WSNs, the epidemiological dynamic is generally used in the study of security issues in WSNs. The applications of epidemiological dynamics are mainly the model’s stability analysis. Recent related studies are shown in Table 1.

However, there are few studies on the application of epidemic dynamics in WRSNs, and there is almost no research on the pulse charging characteristic of WRSNs. Due to the similarity between the propagation characteristic of epidemic disease in the population and the propagation characteristic of malware attack in WRSNs, the application of pulse effect also gives us a novel method to suppress the spreading of malware. Recent related studies are shown in Table 2.

Due to the rechargeable characteristic, the time of the charging behavior relative to the whole process of the spreading of malware [33] is short, and the charging behavior can be thought of as a pulse activity to some extent. The problem of malware spreading under pulse charging is different from that under continuous charging mode [34]. In this paper, inspired by previous works [35] and taking into account the pulse-charging process, a time-delay free model of WRSNs based on pulse charging, SIALS-P (sensitive—infection—anti-malware—low energy sensitive) is proposed. The model reveals the hardware attack process of malware and the pulse charging process in WRSNs. In this paper, we introduce the local and global stability of the malware-free *T*-period solution of SIALS-P by using stability analysis theory to prove the persistence of malware transmission and propose an optimal impulsive control strategy.

The main contents of this paper are as follows: the modeling of SIALS-P and the proof of the existence of a malware-free equilibrium state and malware-free *T*-period solution will be introduced in Section 2. Section 3 prove the local stability and global stability of the system. Section 4 demonstrates the persistence of disease. Section 5 proposes and proves the optimal impulsive control strategy. Section 6 shows the simulation results. Section 7 is the conclusion of this paper.

## 2. Modeling Analysis

### 2.1. Epidemic Modeling on WRSNs

WRSNs is composed of randomly distributed rechargeable nodes. The SIALS (susceptible, infected, anti-malware, low-energy, susceptible) model is first introduced here. It is assumed that the number of nodes increases at a rate Λ, where Λ is greater than 0. The nodes in the network belong to one of six possible compartments. This model describes the relationships among susceptible nodes (*S*), infected nodes (*I*), anti-malware nodes (*A*), low-energy and susceptible nodes (*LS*), low-energy and infected nodes (*LI*) and dysfunctional nodes (*D*). *S* nodes are vulnerable to malware, and *I* nodes are the nodes infected by malware nodes. *A* nodes clear the malware by activating anti-malware; both *LS* and *LI* nodes are at low energy levels and remain dormant. *D* nodes are totally out of function.

According to the knowledge of epidemic dynamics, the epidemiological coefficients of the models are not less than zero [36].

Thus, the dynamical system can be obtained:

(1) $$\left\{\begin{array}{l} \frac{dS\left(t\right)}{dt}=\Lambda -\left(\alpha_{1}I\left(t\right)+\beta_{1}+\mu \right)S\left(t\right),\;\\ \frac{dI\left(t\right)}{dt}=\alpha_{1}S\left(t\right)I\left(t\right)-\left(\alpha_{2}+\beta_{3}+\mu +\alpha \right)I\left(t\right),\\ \frac{dA\left(t\right)}{dt}=-\left(\beta_{2}+\mu \right)A\left(t\right)+\alpha_{2}I\left(t\right),\\ \frac{dLI\left(t\right)}{dt}=-\mu LI\left(t\right)+\beta_{3}I\left(t\right),\\ \frac{dLS\left(t\right)}{dt}=-\mu LS\left(t\right)+\beta_{1}S\left(t\right)+\beta_{2}A\left(t\right),\\ \frac{dD\left(t\right)}{dt}=\mu N\left(t\right)+aI\left(t\right),\; \end{array}\right.$$

where Λ is the birth rate of susceptible nodes, $\alpha_{1}$ is the transmission rate of infected nodes, $\beta_{1}$ is the wastage rate of susceptible nodes becoming low-energy susceptible nodes, μ is the mortality rate of nodes, $\alpha_{2}$ is the clearance rate of anti-malicious nodes to infected nodes, $\beta_{3}$ is the wastage rate of infected nodes becoming low-energy infected nodes, α is the mortality rate of infected nodes, $\beta_{2}$ is the attrition rate of anti-malicious nodes becoming low-power and susceptible nodes.

We have $N\left(t\right)=S\left(t\right)+I\left(t\right)+A\left(t\right)+LS\left(t\right)+LI\left(t\right)$, so the supplementary equation is:

(2) $$\frac{dN\left(t\right)}{dt}=\Lambda -\mu N\left(t\right)-aI\left(t\right),$$

As *t*→∞, the feasible region is governed by $LS\left(t\right)=N\left(t\right)-S\left(t\right)-A\left(t\right)-I\left(t\right)-LI\left(t\right)$:

(3) $$\Omega =\left\{\left(S,A,I,LI\right)\in R^{4}|0\leq N\leq \frac{\Lambda -aI}{\mu }\right\}.\;$$

### 2.2. A Pulse Charging Model

By introducing the pulse charging into the above SIALS model, SIALS-P can be obtained when $t=nT\;\left(n=1,2,3...\right)$:

(4) $$\left\{\begin{array}{l} S\left(t^{+}\right)=S\left(t\right)+\gamma LS\left(t\right),\;\\ I\left(t^{+}\right)=I\left(t\right)+\gamma LI\left(t\right),\\ A\left(t^{+}\right)=A\left(t\right),\\ LI\left(t^{+}\right)=\left(1-\gamma \right)LI\left(t\right),\\ LS\left(t^{+}\right)=\left(1-\gamma \right)LS\left(t\right),\\ N\left(t^{+}\right)=N\left(t\right), \end{array}\right.$$

where T is the pulse charging period, and γ is the charging rate. $nT^{+}$ is used to represent the next instant of nT; that is, pulse charging is to charge the low-energy nodes (LS,LI) at a series of time points. When $t\;\neq nT\left(n=1,2,3...\right)$:

(5) $$\left\{\begin{array}{l} \frac{dS\left(t\right)}{dt}=\Lambda -\left(\alpha_{1}I\left(t\right)+\beta_{1}+\mu \right)S\left(t\right),\\ \frac{dI\left(t\right)}{dt}=\alpha_{1}S\left(t\right)I\left(t\right)-\left(\alpha_{2}+\beta_{3}+\mu +\alpha \right)I\left(t\right),\\ \frac{dA\left(t\right)}{dt}=-\left(\beta_{2}+\mu \right)A\left(t\right)+\alpha_{2}I\left(t\right),\\ \frac{dLI\left(t\right)}{dt}=-\mu LI\left(t\right)+\beta_{3}I\left(t\right),\\ \frac{dLS\left(t\right)}{dt}=-\mu LS\left(t\right)+\beta_{1}S\left(t\right)+\beta_{2}A\left(t\right),\\ \frac{dN\left(t\right)}{dt}=\Lambda -\mu N\left(t\right)-aI\left(t\right), \end{array}\right.$$

The malware-free T-period solution is the periodic solution of T that satisfies the above system of equations when I=0, LI=0 and A=0, where

(6) $$S\left(t\right)+LS\left(t\right)=N\left(t\right)=N\left(\infty \right)=\frac{\Lambda }{\mu },$$

Thus, combined with Equations (4)–(6), we can obtain:

(7) $$\left.\begin{array}{c} \frac{dS\left(t\right)}{dt}=\Lambda -\left(\beta_{1}+\mu \right)S\left(t\right),\;\;\;\;\\ \frac{dLS\left(t\right)}{dt}=-\mu LS\left(t\right)+\beta_{1}S\left(t\right), \end{array}\right\}\;t\neq nT(n=1,2,3\ldots ),\;\left.\begin{array}{c} S\left(t^{+}\right)=\left(1-\gamma \right)S\left(t\right)+\frac{\Lambda \gamma }{\mu },\\ LS\left(t^{+}\right)=\left(1-\gamma \right)LS\left(t\right),\;\;\;\;\;\;\; \end{array}\right\}\;t=nT(n=1,2,3\ldots ).$$

In the time interval $\left[nT,\left(n+1\right)T\right]$, the integral of Equation (7) in the period of two concurrent pulses can be obtained:

(8) $$LS\left(t\right)=\frac{\beta_{1}\Lambda }{\left(\beta_{1}+\mu \right)\mu }+\left(LS\left(nT^{+}\right)-\frac{\beta_{1}\Lambda }{\left(\beta_{1}+\mu \right)\mu }\right)e^{-(\beta_{1}+\mu )\left(t-nT\right)},$$

where $LS\left(nT\right)$ is the initial value at the nth pulse time. Using the stroboscopic map [37], in other words, the pulse charging cycle is taken as the stroboscope sampling interval. In the nth pulse charging cycle, the value of the state variable at the initial moment of the pulse charging cycle is used to represent the value at the end of the pulse charging cycle. Therefore, we have $LS_{n+1}=f\left(LS_{n}\right)$ when $LS_{n+1}=LS\left(\left(n+1\right)T^{+}\right)$. Thus, the relationship between different cycles can be obtained:

(9) $$LS_{n+1}=\left(1-\gamma \right)\left[\frac{\beta_{1}\Lambda }{\left(\beta_{1}+\mu \right)\mu }+\left(LS_{n}-\frac{\beta_{1}\Lambda }{\left(\beta_{1}+\mu \right)\mu }\right)e^{-(\beta_{1}+\mu )T}\right],$$

When the equilibrium state is reached, there is $LS_{n+1}=LS_{n}$ between the two cycles, and the equilibrium state can be obtained:

(10) $$LS^{*}=\frac{\frac{\beta_{1}\Lambda }{\left(\beta_{1}+\mu \right)\mu }\left(1-\gamma \right)\left(e^{(\beta_{1}+\mu )T}-1\right)}{e^{(\beta_{1}+\mu )T}-1+\gamma },$$

When t→∞, from Equation (6) we have:

(11) $$S^{*}=\frac{\Lambda }{\mu }-LS^{*},\;$$

The malware-free *T*-period solution can be obtained:

(12) $$\left\{\begin{array}{c} \tilde{LS}\left(t\right)=\frac{\beta_{1}\Lambda }{\left(\beta_{1}+\mu \right)\mu }+\left(LS^{*}-\frac{\beta_{1}\Lambda }{\left(\beta_{1}+\mu \right)\mu }\right)e^{-(\beta_{1}+\mu )\left(t-nT\right)},\\ \tilde{S}\left(t\right)=\frac{\wedge }{\mu }-\tilde{LS}\left(t\right). \end{array}\right.$$

where $t\;\in \;\left[nT,\left(n+1\right)T\right]$.$\;\left(\tilde{S}\left(t\right),\;\tilde{LS}\left(t\right)\right)$ is the malware-free T-period solution of Equation (7).

## 3. Stability Analysis

The basic reproduction number $R_{0}$ is an important parameter in the early stage of malicious virus infection. It represents the expectation of the number of susceptible nodes that can be infected by an infected node in its average infection cycle after an infected node is introduced into the susceptible node. In general, $R_{0}=1$ can be used as a threshold to determine whether malware is dead or not.

**Theorem** **1.** *The equilibrium states*$S^{*}$and$LS^{*}$of Equations (10) and (11) are locally asymptotically stable and globally asymptotically stable if$R_{0}<1$.

**Proof of Theorem** **1.** Taking the equilibrium states $S^{*}$ and $LS^{*}$ as the initial value of Equation (9), we have:

(13) $${\left|\frac{df\left(LS_{n}\right)}{dLS}\right|}_{LS=LS^{*}}<1$$

Therefore, the local stability of the equilibrium state $LS^{*}$ of Equation (10) is locally asymptotically stable according to the stability criterion of differential systems [38]. Because $S^{*}=\frac{\Lambda }{\mu }-LS^{*}$, the local stability of the equilibrium state $S^{*}$ is locally asymptotically stable.

The local stability of $S^{*}$ and $LS^{*}$ means the global stability of $S^{*}$ and $LS^{*}$. This implies that the pulse immunization operations produce the sequences of $S_{n}$ and $LS_{n}$ that converge to the equilibrium states $S^{*}$ and $LS^{*}$. □

For the stability of a malware-free *T*-period solution of Equation (7), we conclude the following theorems:

**Theorem** **2.** *The malware-free T-period solution (*$\tilde{S}\left(t\right),0,0,0,\;\tilde{LS}\left(t\right)$*) of the system, is locally asymptotically stable if*$R_{0}<1$.

**Proof of Theorem** **2.** Let $Q\left(t\right)$ be an *n* × *n* matrix. $\Phi \left(t\right)$ is the basic solution matrix of the linear differential system $x’\left(t\right)=Q\left(t\right)x\left(t\right)$. In addition, let $r\left(\Phi Q\left(t\right)\right)$ be the spectral radius of $\Phi Q\left(t\right)$. Using Floquet theory [39], the local stability of the malware-free T-period solution $\tilde{(S}\left(t\right),0,0,0,\;\tilde{LS}\left(t\right))$ can be proven. Let the disturbance of the malware-free T-period solution of the system be:

(14) $$x\left(t\right)=\left(S\left(t\right),I\left(t\right),A\left(t\right),LI\left(t\right),LS\left(t\right)\right),$$

Through the linear approximation of Equations (4)–(6), we can obtain the following impulsive differential equation:

(15) $$\left\{\begin{array}{c} x^{\prime }\left(t\right)=Q\left(t\right)x\left(t\right),\;\;\;\;\;\;\;\;\;\;\;t\neq nT,n\in N,\\ x\left(t^{+}\right)=Px\left(t\right),\;\;\;\;\;\;\;\;\;\;\;\;\;\;\;\;t=nT,n\in N, \end{array}\right.$$

where

(16) $$Q\left(t\right)=\left[\begin{array}{cc} U & B\\ 0 & F-V \end{array}\right],\;\;\;\;\;\;\;P=\left[\begin{array}{cc} P1 & 0\\ 0 & P2 \end{array}\right],\;U=\left[\begin{array}{ccc} -\left(\beta_{1}+\mu \right) & 0 & 0\\ 0 & -\left(\beta_{2}+\mu \right) & 0\\ \beta_{1} & \beta_{2} & -\mu \end{array}\right],\;\;\;\;\;\;\;B=\left[\begin{array}{cc} -\alpha_{1}S^{*}\left(t\right) & 0\\ \alpha_{2} & 0\\ 0 & 0 \end{array}\right],\;F=\left[\begin{array}{cc} \alpha_{1}S^{*}\left(t\right) & 0\\ 0 & 0 \end{array}\right],\;\;\;\;\;\;\;\;\;\;V=\left[\begin{array}{cc} \alpha_{2}+\beta_{3}+\mu +a & 0\\ -\beta_{3} & \mu \end{array}\right],\;P_{1}=\left[\begin{array}{ccc} 1 & 0 & \gamma \\ 0 & 1 & 0\\ 0 & 0 & 1-\gamma \end{array}\right],\;\;\;\;\;\;\;\;P_{2}=\left[\begin{array}{cc} 1 & \gamma \\ 0 & 1-\gamma \end{array}\right],$$

Because $\Phi_{Q}\left(t\right)=\left[\Phi_{i_{j}}\right]$ is the basic solution matrix of the linear differential system $x’\left(t\right)=Q\left(t\right)x\left(t\right)$, then $\dot{\Phi_{\;}}\left(t\right)=\Phi_{\;}\left(t\right)Q\left(t\right)$, where $\Phi_{\;}\left(0\right)=E_{0}$ ($E_{0}$ is the unit moment matrix). The differential equation is solved, and we have:

(17) $$\Phi \left(t\right)=\left(\begin{array}{cc} e^{UT} & \Phi_{B}\left(t\right)\\ 0 & \Phi_{F-V}\left(t\right) \end{array}\right),$$

when t=nT, it is easy to figure out:

(18) $$P\Phi \left(t\right)=\left(\begin{array}{cc} P_{1}e^{UT} & P_{1}\Phi_{B}\left(t\right)\\ 0 & P_{2}\Phi_{F-V}\left(t\right) \end{array}\right),$$

(19) $$P_{1}e^{UT}=\left[\begin{array}{ccc} e^{-\left(\beta_{1}+\mu \right)T}+\gamma \beta_{1}Te^{-\mu T}\; & \gamma \beta_{2}Te^{-\mu T} & \gamma e^{-\mu T}\\ 0 & e^{-\left(\beta_{2}+\mu \right)T} & 0\\ \left(1-\gamma \right)\beta_{1}Te^{-\mu T}\; & \;\;\;\left(1-\gamma \right)\beta_{2}Te^{-\mu T} & \;\;\;\;\left(1-\gamma \right)e^{-\mu T} \end{array}\right].$$

(20) $$P_{2}\Phi_{F-V}\left(t\right)=\left[\begin{array}{cc} e^{\int_{0}^{T}\left(\alpha_{1}S^{*}\left(t\right)-\left(\alpha_{2}+\beta_{3}+\mu +a\right)\right)dt}+\gamma e^{\beta_{3}T} & \gamma e^{-\mu T}\\ \left(1-\gamma \right)e^{\beta_{3}T} & \left(1-\gamma \right)e^{-\mu T} \end{array}\right].$$

Obviously, the Floquet multipliers of Equation (18) are as follows:

(21) $$\left\{\begin{array}{c} \omega_{1}=r(P_{1}e^{UT}),\\ \omega_{2}=r(P_{2}\Phi_{F-V}\left(t\right)), \end{array}\right.$$

According to Floquet theory and pulse differential equation theory [37], the malware-free T-period solution is locally asymptotically stable if $\omega_{i}<1$, where i=1,2. It is difficult for us to see the values of $\omega_{1}$ and $\omega_{2}$ directly. Therefore, in the case that $\omega_{1}$ is less than 1, there exists a threshold, which can be defined as:

(22) $$R_{0}≜r(P_{2}\Phi_{F-V}\left(t\right))$$

According to Floquet theory, when $R_{0}<1$, the malware-free T-period solution ($\tilde{S}\left(t\right),0,0,0,\;\tilde{LS}\left(t\right)$) of the system is locally asymptotically stable. Locally asymptotic stability is only for the domain where the periodic solutions are small, and the asymptotic stability of solutions with arbitrary initial values in region Ω will be proven next. □

**Theorem** **3.** *When* t→∞ *,* $S\left(t\right)\to \tilde{S}\left(t\right)$ *and* $LS\left(t\right)\;\to \tilde{LS}\left(t\right)$ *, the malware-free T-period solution of the system is globally asymptotically stable.*

**Proof of Theorem** **3.** From Equation (2), we have: $\Lambda -\left(\mu +a\right)N\left(t\right)\;\leq \;\frac{dN\left(t\right)}{dt}\;\leq \;\Lambda -\mu N\left(t\right)$. It follows that

(23) $$\frac{\Lambda }{\mu +a}\leq \underset{t\to \infty }{\lim}inf\;N\left(t\right)\leq \underset{t\to \infty }{\lim}sup\;N\left(t\right)\leq \frac{\Lambda }{\mu },$$

From Equation (23), we have:

(24) $$S\left(t\right)+LS\left(t\right)\leq \underset{t\to \infty }{\lim}\sup N\left(t\right)\leq \frac{\Lambda }{\mu },$$

From Equations (7) and (24), we can obtain:

(25) $$\left.\begin{array}{c} \frac{dS\left(t\right)}{dt}\leq \Lambda -\left(\beta_{1}+\mu \right)S\left(t\right),\\ \frac{dLS\left(t\right)}{dt}=-\mu LS\left(t\right)+\beta_{1}S\left(t\right)\leq -\left(\mu +\beta_{1}\right)LS\left(t\right)+\frac{\beta_{1}\Lambda }{\mu }, \end{array}\right\}\;t\neq nT,n\in N,\;\left.\begin{array}{c} S\left(t^{+}\right)=\left(1-\gamma \right)S\left(t\right)+\frac{\Lambda \gamma }{\mu },\\ LS\left(t^{+}\right)=\left(1-\gamma \right)LS\left(t\right), \end{array}\right\}\;t=nT,n\in N.$$

Consider the following comparison system:

(26) $$\left.\begin{array}{c} x_{1}^{’}\left(t\right)=\Lambda -\left(\beta_{1}+\mu \right)x_{1}\left(t\right),\\ x_{2}^{’}\left(t\right)=-\left(\mu +\beta_{1}\right)x_{2}\left(t\right)+\frac{\beta_{1}\Lambda }{\mu }, \end{array}\right\}\;t\neq nT,n\in N,\;\left.\begin{array}{c} x_{1}\left(t^{+}\right)=\left(1-\gamma \right)x_{1}\left(t\right)+\frac{\Lambda \gamma }{\mu },\\ x_{2}\left(t^{+}\right)=\left(1-\gamma \right)x_{2}\left(t\right), \end{array}\right\}\;t=nT,n\in N.$$

Through the comparison theorem of impulsive differential equations [40], it can be obtained that $S\left(t\right)\leq x\left(t\right)$ and $LS\left(t\right)\leq x\left(t\right)$. When t→∞, we can obtain $x_{1}\left(t\right)\to S\left(t\right)$ and $x_{2}\left(t\right)\to LS\left(t\right)$. Then, there are $\varepsilon >0\;\mathrm{and}\;t_{1}>0$. For any time $t>t_{1}$, we have:

(27) $$S\left(t\right)\leq x_{1}\left(t\right)<\tilde{S}\left(t\right)+\varepsilon ,LS\left(t\right)\leq x_{2}\left(t\right)<\tilde{LS}\left(t\right)+\varepsilon ,$$

(28) $$\left.\begin{array}{c} \frac{dI\left(t\right)}{dt}\leq \left[-\alpha_{2}-\mu -a-\beta_{3}+\alpha_{1}\left(\tilde{S}\left(t\right)+\varepsilon \right)\right]I\left(t\right),\\ \frac{dLI\left(t\right)}{dt}\leq -\mu LI\left(t\right)+\beta_{3}I\left(t\right), \end{array}\right\}\;t\neq nT,n\in N,\;\left.\begin{array}{c} I\left(t^{+}\right)=I\left(t\right)+\gamma LI\left(t\right),\\ LI\left(t^{+}\right)=\left(1-\gamma \right)LI\left(t\right), \end{array}\right\}\;t=nT,n\in N.$$

Suppose $u\left(t\right)=\left(\begin{array}{c} u_{1}\left(t\right)\\ u_{2}\left(t\right) \end{array}\right)$, we have:

(29) $$\begin{array}{ll} \left(F-V\right)u\left(t\right) & =\left(\begin{array}{cc} -\alpha_{2}-\mu -a-\beta_{3}+\alpha_{1}\left(\tilde{S}\left(t\right)+\varepsilon \right) & 0\\ \beta_{3} & -\mu \end{array}\right)\left(\begin{array}{c} u_{1}\left(t\right)\\ u_{2}\left(t\right) \end{array}\right)\\ & =\left(\begin{array}{c} \left[-\alpha_{2}-\mu -a-\beta_{3}+\alpha_{1}\left(\tilde{S}\left(t\right)+\varepsilon \right)\right]u_{1}\left(t\right)\\ -\mu u_{2}\left(t\right)+\beta_{3}u_{1}\left(t\right) \end{array}\right), \end{array}$$

Consider the following comparison system:

(30) $$u^{\prime }\left(t\right)=\left(F-V\right)u\left(t\right),t\neq nT,n\in N,\;\left.\begin{array}{c} u_{1}\left(t^{+}\right)=u_{1}\left(t\right)+\gamma u_{2}\left(t\right),\;\\ u_{2}\left(t^{+}\right)=\left(1-\gamma \right)u_{2}\left(t\right),\;\; \end{array}\right\}t=nT,n\in N.$$

The solution of the system can be expressed as:

(31) $$u\left(u_{1},u_{1}\right)=\Phi_{F-V}\left(t-nT\right)u\left(nT^{+}\right),$$

when t=nT, $u\left(\left(n+1\right)T^{+}\right)=P_{2}\Phi_{F-V}\left(t-nT\right)u\left(nT^{+}\right).$ When t→∞, $u_{1}\to 0$ and $u_{2}\to 0$. At this time, $\underset{t\to \infty }{\lim}I\left(t\right)=0\;\mathrm{and}\;\underset{t\to \infty }{\lim}LI\left(t\right)=0$. Then, there are $\varepsilon_{2}>0\;\mathrm{and}\;t_{2}>t_{1}$. For any $t>t_{2}$, we have $0<I\left(t\right)<\varepsilon_{2}$, $0<A\left(t\right)<\varepsilon_{2}$ and $0<LI\left(t\right)<\varepsilon_{2}$.

Thus, from Equations (7) and (24), we have:

(32) $$\left.\begin{array}{c} \Lambda -\left(\alpha_{1}\varepsilon_{2}+\mu +\beta_{1}\right)S\left(t\right)\leq \frac{dS\left(t\right)}{dt}\leq \Lambda -\left(\mu +\beta_{1}\right)S\left(t\right),\\ -\Lambda +\left(\mu +\beta_{1}\right)S\left(t\right)\leq \frac{dLS\left(t\right)}{dt}\leq -\left(\mu +\beta_{1}\right)LS\left(t\right)+\frac{\beta_{1}\Lambda }{\mu }+\beta_{2}\varepsilon_{2}, \end{array}\right\}\;t\neq nT,n\in N,\;\left.\begin{array}{c} S\left(t^{+}\right)=S\left(t\right)+\gamma LS\left(t\right),\\ LS\left(t^{+}\right)=\left(1-\gamma \right)LS\left(t\right), \end{array}\right\}\;t=nT,n\in N.$$

Then, consider the following comparison system:

(33) $$\left.\begin{array}{c} y_{1}^{’}t=\Lambda -\left(\alpha_{1}\varepsilon_{2}+\mu +\beta_{1}\right)y_{1}\left(t\right),\\ y_{2}^{’}t=-\Lambda +\left(\mu +\beta_{1}\right)S\left(t\right), \end{array}\right\}\;t\neq nT,n\in N,\;\left.\begin{array}{c} y_{1}\left(t^{+}\right)=S\left(t\right)+\gamma LS\left(t\right),\\ y_{2}\left(t^{+}\right)=\left(1-\gamma \right)y_{2}t, \end{array}\right\}\;t=nT,n\in N.$$

Similar to the processing methods for Equations (4) and (5), a set of positive solutions $\tilde{y}=\left(\tilde{y_{1}},\tilde{y_{2}}\right)$ that are globally asymptotically stable for the comparison system can be obtained, and $\underset{\varepsilon_{2}\to 0}{\lim}\tilde{y}=\left(\tilde{S}\left(t\right),\tilde{LS}\left(t\right)\right)$. Through the comparison theorem of impulsive differential equations, we can obtain:

(34) $$\left\{\begin{array}{c} y_{1}t<S\left(t\right)<x_{1}t,\\ y_{2}t<LS\left(t\right)<x_{2}t, \end{array}\right.$$

when t→∞, we have:

(35) $$\left\{\begin{array}{c} y_{1}\to \tilde{y_{1}},x_{1}\to \tilde{S}\left(t\right),\\ y_{2}\to \tilde{y_{2}},x_{2}\to \tilde{LS}\left(t\right), \end{array}\right.$$

Then, there is $t_{3}>t_{2}$. For $\varepsilon_{3}>0,$ which is small enough, we can obtain when $t>t_{3}:$

(36) $$\tilde{y_{1}}-\varepsilon_{3}<S\left(t\right)<\tilde{S}\left(t\right)+\varepsilon_{3},\tilde{y_{2}}-\varepsilon_{3}<LS\left(t\right)<\tilde{LS}\left(t\right)+\varepsilon_{3}.$$

Therefore, when t→∞, $S\left(t\right)\to \tilde{S}\left(t\right)$ and $LS\left(t\right)\to \tilde{LS}\left(t\right)$. Therefore, the malware-free *T*-period solution of the system is globally attractive. That is, after a certain amount of time, the solution is absorbed into a bounded set through motion. □

## 4. Persistence of Malware Transmission

In this section, the persistence of malware transmission will be discussed. If certain conditions are met, the malware will not die out; that is, the spread of malware in WRSNs will continue, which is the persistence of malware transmission. The infected nodes (I noses) are the determinants of the persistence of malware transmission. In order to obtain this result, the following is given:

**Lemma** **1.** *When* $\;R_{0}>1$ *, there is a positive number* δ *to make the solution of the SIALS-P to satisfy the following inequality:*

(37) $$\left\{\begin{array}{c} \underset{t\to \infty }{\lim}sup\;I\left(t\right)>\delta ,\\ \underset{t\to \infty }{\lim}sup\;LI\left(t\right)>\delta . \end{array}\right.$$

**Proof of Lemma** **1.** Using the proof by contradiction, if the above conclusions are not established, there is $t_{1}>0$, and for any $t>t_{1}$, we have $I\;\left(t\right)<\delta$. From Equations (4) and (5), we can obtain:

(38) $$\left.\begin{array}{c} \frac{dS\left(t\right)}{dt}\geq \Lambda -\left(\alpha_{1}\delta +\beta_{1}+\mu \right)S\left(t\right),\\ \frac{dLS\left(t\right)}{dt}\;\geq -\Lambda +\left(\mu +\beta_{1}\right)S\left(t\right)+\beta_{2}A\left(t\right), \end{array}\right\}\;t\neq nT,n\in N,\;\left.\begin{array}{c} S\left(t^{+}\right)=S\left(t\right)+\gamma LS\left(t\right),\\ LS\left(t^{+}\right)=\left(1-\gamma \right)LS\left(t\right), \end{array}\right\}\;t=nT,n\in N.$$

Consider the following comparison system:

(39) $$\left.\begin{array}{c} z_{1}^{’}t=\Lambda -\left(\alpha_{1}\delta +\beta_{1}+\mu \right)z_{1}\left(t\right),\;\\ z_{2}^{’}t=-\Lambda +\left(\mu +\beta_{1}\right)z_{1}\left(t\right)+\beta_{2}A\left(t\right), \end{array}\right\}\;t\neq nT,n\in N,\;\left.\begin{array}{c} z_{1}^{’}\left(t^{+}\right)=z_{1}\left(t\right)+\gamma z_{2}\left(t\right),\\ z_{2}^{’}\left(t^{+}\right)=\left(1-\gamma \right)z_{2}\left(t\right), \end{array}\right\}\;t=nT,n\in N.$$

From the comparison theorem. We can obtain:

(40) $$\begin{array}{c} St\geq z_{1}\left(t\right),\;\\ LS\left(t\right)\geq z_{2}\left(t\right). \end{array}$$

The positive periodic solution $\tilde{Z}=({\tilde{Z}}_{1},{\tilde{Z}}_{2})$ of Equation (39) which is globally stable, can be obtained and $\underset{\delta \to 0}{lim}\tilde{Z}=\left(\tilde{S},\tilde{LS}\right)$. Therefore, there is a positive number $\delta_{1}$ and for any $\varepsilon_{1}>0$ and $\delta <\delta_{1}$, we have ${\tilde{Z}}_{1}\geq \tilde{S}-\varepsilon_{1}$ and $z_{2}^{*}\geq LS^{*}-\varepsilon_{1}$. From the comparison theorem of impulsive differential equations, we have a time variable $t_{2}>t_{1}$ and a positive number $\varepsilon_{2}>0$. For any time $t>t_{2}$, the inequality system is as follows:

(41) $$\begin{array}{c} St\geq z_{1}\left(t\right)\geq {\tilde{Z}}_{1}-\varepsilon_{2}\geq \tilde{S}-\varepsilon_{1}-\varepsilon_{2},\;\;\\ LSt\geq z_{2}\left(t\right)\geq {\tilde{Z}}_{2}-\varepsilon_{2}\geq \tilde{LS}-\varepsilon_{1}-\varepsilon_{2}. \end{array}$$

The inequality system of Equation (41) is substituted into Equations (4) and (5). We can obtain:

(42) $$\left.\begin{array}{c} \;\;\frac{dI\left(t\right)}{dt}\geq \left[-\alpha_{2}-\mu -a-\beta_{3}+\alpha_{1}\left(S^{*}-\varepsilon_{1}-\varepsilon_{2}\right)\right]I\left(t\right),\\ \frac{dLI\left(t\right)}{dt}\geq -\mu LI\left(t\right)+\beta_{3}I\left(t\right), \end{array}\right\}\;t\neq nT,n\in N,\;\left.\begin{array}{c} I\left(t^{+}\right)=I\left(t\right)+\gamma LI\left(t\right),\\ LI\left(t^{+}\right)=\left(1-\gamma \right)LI\left(t\right), \end{array}\right\}\;t=nT,n\in N.$$

If $\varepsilon_{1}$ and $\varepsilon_{2}$ are small enough to approach 0, the system can be simplified as:

(43) $$\left.\begin{array}{c} \frac{dI\left(t\right)}{dt}\geq \left[-\alpha_{2}-\mu -a-\beta_{3}+\alpha_{1}S^{*}\right]I\left(t\right),\\ \frac{dLI\left(t\right)}{dt}\geq -\mu LI\left(t\right)+\beta_{3}I\left(t\right), \end{array}\right\}\;t\neq nT,n\in N,\;\left.\begin{array}{c} I\left(t^{+}\right)=I\left(t\right)+\gamma LI\left(t\right),\\ LI\left(t^{+}\right)=\left(1-\gamma \right)LI\left(t\right), \end{array}\right\}\;t=nT,n\in N.$$

Through the comparison theorem, let $u\left(t\right)=\left(\begin{array}{c} u_{1}\left(t\right)\\ u_{2}\left(t\right) \end{array}\right)$ and construct the following system:

(44) $$u^{\prime }\left(t\right)=\left(F-V\right)u\left(t\right),t\neq nT,n\in N,\;\left.\begin{array}{c} u_{1}\left(t^{+}\right)=u_{1}\left(t\right)+\gamma u_{2}\left(t\right),\;\;\;\;\\ u_{2}\left(t^{+}\right)=\left(1-\gamma \right)u_{2}\left(t\right),\;\;\;\;\; \end{array}\right\}t=nT,n\in N.$$

The above system satisfies $u\left(t,nT,u\left(nT^{+}\right)\right)=\phi_{F-v}\left(t-nT\right)u\left(nT^{+}\right)$, $u\left(\left(n+1\right)T^{+}\right)=P_{2}\phi_{F-v}\left(t-nT\right)u\left(nT^{+}\right)$. When $R_{0}>1$, as t→∞ and $u_{1}\to \infty$ and $u_{2}\to \infty$, we can obtain the conclusion as follows:

(45) $$\begin{array}{c} \underset{t\to \infty }{\lim}I\left(t\right)=\infty ,\\ \underset{t\to \infty }{\lim}LI\left(t\right)=\infty . \end{array}$$

The above conclusion contradicts the condition It<δ and $LI\left(t\right)<\delta$ which was established previously. Hence, Lemma 1 is proved. □

Through Lemma 1, we can obtain the following theorem:

**Theorem** **4.** *When* $R_{0}>1$ *, the malware transmission is uniformly persistent, that is, there is a positive number* η *that makes the solution of the system satisfy the following inequality:*

(46) $$\begin{array}{c} \underset{t\to \infty }{\lim}inf\;I\left(t\right)>\eta ,\;\\ \underset{t\to \infty }{\lim}inf\;LI\left(t\right)>\eta . \end{array}$$

**Proof of Theorem** **4.** According to Lemma 1, there are two possible situations when the malware transmission is uniformly persistent:

(I). When the time variable T is large enough, $I\left(t\right)>\eta$ and $LI\left(t\right)>\eta ;$

(II). When the time variable T is large enough, $I\left(t\right)$ and $LI\left(t\right)$ oscillate nearby η.

If case (I) is true, it is clear that malware transmission is uniformly persistent. Therefore, we focus on case (II). From Lemma 1, we can obtain:

(47) $$\begin{array}{c} \underset{t\to \infty }{\lim}sup\;I\left(t\right)>\delta ,\\ \underset{t\to \infty }{\lim}sup\;LI\left(t\right)>\delta . \end{array}$$

Therefore, in the case of oscillation, we have:

(48) $$\left.\begin{array}{c} I\left(t_{1}\right)\geq \delta ,\\ LI\left(t_{1}\right)\geq \delta , \end{array}\right\}\;t_{1}\in \left(n_{1}T,\left(n+1\right)T\right],\;\left.\begin{array}{c} \;I\left(t_{2}\right)\geq \delta ,\\ LI\left(t_{2}\right)\geq \delta , \end{array}\right\}\;t_{2}\in \left(n_{2}T,\left(n+1\right)T\right].$$

where $n_{2}>n_{1}$ and when $t\in \left[t_{1},t_{2}\right]$, it can be obtained as follows:

(49) $$\frac{dLI\left(t\right)}{dt}=-\mu LI\left(t\right)+\beta_{3}I\left(t\right)\geq -\mu LI\left(t\right),\;\;\;\;t\neq nT,n\in N,\;LI\left(t^{+}\right)=\left(1-\gamma \right)LI\left(t\right),\;\;\;\;\;\;\;t=nT,n\in N.$$

We can obtain:

(50) $$LI\left(t\right)\geq LI\left(t_{1}\right)e^{-\mu \left(t-t_{1}\right)}\geq \delta e^{-\mu \left(t-t_{1}\right)}\geq \delta e^{-\mu \left(n_{2}-n_{1}+1\right)T},$$

Additionally, when t=nT, we can obtain:

(51) $$LI\left(t\right)\geq \delta {\left(1-\gamma \right)}^{n_{2}-n_{1}}e^{-\mu \left(n_{2}-n_{1}+1\right)T},$$

Then, for $I\left(t\right)$, we have:

(52) $$\frac{dI\left(t\right)}{dt}=\left[-\alpha_{2}-\mu -a-\beta_{3}+\alpha_{1}S\left(t\right)\right]I\left(t\right)\geq \left(-\alpha_{2}-\mu -a-\beta_{3}\right)I\left(t\right),\;I\left(t^{+}\right)=I\left(t\right)+\gamma LI\left(t\right).$$

By applying the solution method similar to Equation (52), we have:

(53) $$I\left(t\right)\geq \delta e^{\left(-\alpha_{2}-\mu -a-\beta_{3}\right)\left(n_{2}-n_{1}+1\right)T}+\gamma^{n_{2}-n_{1}}\delta {\left(1-\gamma \right)}^{n_{2}-n_{1}}e^{-\mu \left(n_{2}-n_{1}+1\right)T},$$

Let $\eta_{1}=min\left\{\delta e^{\left(-\alpha_{2}-\mu -a-\beta_{3}\right)\left(n_{2}-n_{1}+1\right)T}+\gamma^{n_{2}-n_{1}}\delta {\left(1-\gamma \right)}^{n_{2}-n_{1}}e^{-\mu \left(n_{2}-n_{1}+1\right)T},\delta {\left(1-\gamma \right)}^{n_{2}-n_{1}}e^{-\mu \left(n_{2}-n_{1}+1\right)T}\right\}.$

Due to $n_{2}-n_{1}\geq 0$ and the fact that it is bounded, $\eta_{1}$ cannot be infinitesimal. Hence, we can obtain $I\left(t\right)\geq \eta_{1}$ and $LI\left(t\right)\geq \eta_{1}.$

For $t>t_{2}$, there is a positive number $\eta_{2}$. Therefore, we can obtain the sequence $\left\{\eta_{j}\right\},\;j=1,2,\ldots k\ldots ,$ where

(54) $$\eta_{1}=min\{\delta e^{\left(-\alpha_{2}-\mu -a-\beta_{3}\right)\left(n_{2}-n_{1}+1\right)T}+\gamma^{n_{2}-n_{1}}\delta {\left(1-\gamma \right)}^{n_{2}-n_{1}}e^{-\mu \left(n_{2}-n_{1}+1\right)T},\;{\left(1-\gamma \right)}^{n_{2}-n_{1}}e^{-\mu \left(n_{2}-n_{1}+1\right)T}\},$$

When $t\in \left(t_{k},t_{k+1}\right),$ the inequality can be derived as follows where $t_{k}\in n_{k}T,\left(n_{k}+1\right)T$ and $t_{k+1}\in n_{k+1}T,\left(n_{k+1}+1\right)T$:

(55) $$\begin{array}{c} I\left(t\right)\geq \eta_{k}>0,\\ LI\left(t\right)\geq \eta_{k}>0. \end{array}$$

Let $\eta^{*}=\min\;\eta_{j}.$ For any $t>t_{1},$ there is $I\left(t\right)\geq \eta^{*}>0$ and $LI\left(t\right)\geq \eta^{*}>0$. Therefore, Theorem 4 is proven. □

## 5. Optimal Control

In the optimal control theory of epidemic dynamics, the primary goal is to minimize the number of infections while minimizing the cost of vaccination [41].

The probability that the infected node becomes the anti-malware node after receiving information from the anti-malware node is $\alpha_{2}$. In the process of constructing the minimum objective function, $\alpha_{2}$ is selected as the control variable that changes over time between the pulse points and is represented by $\alpha_{2}\left(t\right)$, and $0\leq \alpha_{2}\left(t\right)\leq 1$. Control variable $\alpha_{2}\left(t\right)$ is constantly changing over time. Unlike $\alpha_{2}\left(t\right)$, $v\left(nT\right)$ is the control variable of the pulse point and a targeted therapy strategy to reduce infected nodes, and $0\leq v\left(nT\right)\leq 1$.

In this section, the ultimate goal of optimal control is to minimize the number of infected nodes and minimize the cost of activating anti-malware and detecting and killing malware. Thus, in the SIALS-P model, we can construct the minimized objective function as:

(56) $$J\left(g_{i}\left(t\right),v_{i}\left(nT\right)\right)=\int_{0}^{\delta T}(A_{1}I\left(t\right)+A_{2}LI\left(t\right)+\frac{A_{3}}{2}\alpha_{2}^{2}\left(t\right))dt+\overset{\eta }{\underset{i=1}{\sum }}\left(\frac{B}{2}v^{2}\left(nT\right)\right),$$

where δT represents the duration of optimal control and δ∈N. $A_{1}$ and $A_{2}$ are the monitoring costs of I nodes and LI nodes, respectively. $A_{3}$ and B are the costs of implementing control strategies.

Introduce control variables $\alpha_{2}$ and $v\left(nT\right)$ into Equations (4) and (5). The objective function subject is:

(57) $$\left\{\begin{array}{l} \left.\begin{array}{l} \frac{dS\left(t\right)}{dt}=\Lambda -\left(\alpha_{1}I\left(t\right)+\beta_{1}+\mu \right)S\left(t\right),\\ \frac{dI\left(t\right)}{dt}=\alpha_{1}S\left(t\right)I\left(t\right)-\left(\alpha_{2}\left(t\right)+\beta_{3}+\mu +\alpha \right)I\left(t\right)\\ \frac{dA\left(t\right)}{dt}=-\left(\beta_{2}+\mu \right)A\left(t\right)+\alpha_{2}\left(t\right)I\left(t\right),\\ \frac{dLI\left(t\right)}{dt}=-\mu LI\left(t\right)+\beta_{3}I\left(t\right),\\ \frac{dLS\left(t\right)}{dt}=-\mu LS\left(t\right)+\beta_{1}S\left(t\right)+\beta_{2}A\left(t\right),\;\; \end{array}\right\}t\neq nT,\;n\in N\\ \left.\begin{array}{l} S\left(t^{+}\right)=S\left(t\right)+\gamma LS\left(t\right)+v\left(nT\right)I\left(t\right),\\ I\left(t^{+}\right)=I\left(t\right)+\gamma LI\left(t\right)-v\left(nT\right)I\left(t\right),\\ A\left(t^{+}\right)=A\left(t\right),\\ LI\left(t^{+}\right)=\left(1-\gamma \right)LI\left(t\right),\\ LS\left(t^{+}\right)=\left(1-\gamma \right)LS\left(t\right), \end{array}\right\}t=nT,\;n\in N \end{array}\right.$$

In order to achieve the optimal control objective, the Hamiltonian *H* without control as the function is constructed as:

(58) $$H=A_{1}I\left(t\right)+A_{2}LI\left(t\right)+\frac{A_{3}}{2}\alpha_{2}^{2}\left(t\right)+\lambda_{1}\left(t\right)\dot{S_{i}\left(t\right)}+\lambda_{2}\left(t\right)\dot{I_{i}{}_{\;}\left(t\right)}+\lambda_{3}\left(t\right)\dot{A_{\;}\left(t\right)+}\lambda_{4}\left(t\right)\dot{LI_{\;}\left(t\right)+}\lambda_{5}\left(t\right)\dot{LS_{\;}\left(t\right)}$$

We construct the impulse Hamiltonian function $H^{P}$ defined as:

(59) $$H^{P}=\frac{B}{2}v^{2}\left(nT\right)+\gamma \lambda_{1}\left(nT^{+}\right)LS\left(t\right)+v\lambda_{1}\left(nT^{+}\right)I\left(t\right)+\gamma \lambda_{2}\left(nT^{+}\right)LI\left(t\right)-v\lambda_{2}\left(nT^{+}\right)I\left(t\right)-\gamma \lambda_{4}\left(nT^{+}\right)LI\left(t\right)-\gamma \lambda_{5}\left(nT^{+}\right)LS\left(t\right)$$

We use the Pontryagin maximum principle to obtain the necessary conditions for optimal control.

**Theorem** **5.** *Introduce optimal controls*$\alpha_{2}^{*}\left(t\right)$*and*$v^{*}\left(nT\right)$*, and solutions*$S^{*}\left(t\right)$*,*$I^{*}\left(t\right)$*,*$A^{*}\left(t\right)$*,*$LI^{*}\left(t\right)$*and*$LS^{*}\left(t\right)$*into Equation (57). There exist adjoint variables*$\lambda_{k}\left(t\right)$*,*k=1, 2, 3, 4. t≠nT*provides an optimal control*$\alpha_{2}^{*}\left(t\right)$*. The adjoint variables*$\lambda_{k}\left(t\right)$*satisfy the following adjoint differential system.*

(60) $$\left\{\begin{array}{l} \frac{d\lambda_{1}\left(t\right)}{dt}=-\frac{\partial H}{\partial S}=-\left(-\lambda_{1}\left(t\right)\left(\alpha_{1}I\left(t\right)+\beta_{1}+\mu \right)+\alpha_{1}\lambda_{2}\left(t\right)I\left(t\right)+\beta_{1}\lambda_{5}\left(t\right)\right),\\ \frac{d\lambda_{2}\left(t\right)}{dt}=-\frac{\partial H}{\partial I}=-\left(A_{1}+\left(\alpha_{1}S\left(t\right)-(\alpha_{2}\left(t\right)+\beta_{3}+\mu +\alpha \right)\right)\lambda_{2}\left(t\right)+\alpha_{2}\left(t\right)\lambda_{3}+\beta_{3}\lambda_{4}),\\ \frac{d\lambda_{3}\left(t\right)}{dt}=-\frac{\partial H}{\partial A}=-\left(-(\beta_{2}+\mu \;\right)\lambda_{3}+\beta_{2}\lambda_{5}),\\ \frac{d\lambda_{4}\left(t\right)}{dt}=-\frac{\partial H}{\partial LI}=-\left(A_{2}-\mu \lambda_{4}\right),\\ \frac{d\lambda_{5}\left(t\right)}{dt}=-\frac{\partial H}{\partial LS}=-\left(-\mu \lambda_{5}\right), \end{array}\right.$$

Transversality conditions are $\lambda_{k}\left(\delta T\right)=0$, and the optimal control $\alpha_{2}^{*}\left(t\right)$ can be solved as $\frac{\partial H}{\partial \alpha_{2}\left(t\right)}=0$. Thus, we have the optimal control of the continuous part as follows:

(61) $$\alpha_{2}^{*}\left(t\right)=\frac{\left(\lambda_{2}\left(t\right)-\lambda_{3}\left(t\right)\right)I^{*}\left(t\right)}{A_{3}},$$

t=nT provides the impulse optimal control $v^{*}\left(nT\right)$. There exist adjoint variables $\lambda_{k}\left(nT\right)$, k=1, 2, 3, 4 and we have:

(62) $$\left\{\begin{array}{l} \lambda_{1}\left(nT\right)=\lambda_{1}\left(nT^{+}\right),\\ \lambda_{2}\left(nT\right)=\lambda_{2}\left(nT^{+}\right)-v\lambda_{2}\left(nT^{+}\right)+v\lambda_{1}\left(nT^{+}\right),\\ \lambda_{3}\left(nT\right)=\lambda_{3}\left(nT^{+}\right),\\ \lambda_{4}\left(nT\right)=\lambda_{4}\left(nT^{+}\right)-\gamma \lambda_{4}\left(nT^{+}\right)+\gamma \lambda_{2}\left(nT^{+}\right),\\ \lambda_{5}\left(nT\right)=\lambda_{5}\left(nT^{+}\right)-\gamma \lambda_{5}\left(nT^{+}\right)+\gamma \lambda_{1}\left(nT^{+}\right), \end{array}\right.$$

The optimality condition at $v\left(nT\right)$ = $v^{*}\left(nT\right)$ implies that $\frac{\partial H^{P}}{\partial v\left(nT\right)}=0$. Therefore, the optimal control in any impulse point is obtained.

(63) $$v^{*}\left(nT\right)=\frac{\left(\lambda_{2}\left(nT^{+}\right)-\lambda_{1}\left(nT^{+}\right)\right)I^{*}\left(t\right)}{B},$$

$S^{*}\left(t\right)$, $I^{*}\left(t\right)$, $A^{*}\left(t\right)$, $LI^{*}\left(t\right)$ and $LS^{*}\left(t\right)$ are the solutions for Equation (57) to perform optimal control. Let $x\left(t\right)=\left(S\left(t\right),I_{\;}\left(t\right),A_{\;}\left(t\right),LI\left(t\right),LS\left(t\right)\right)$, which is left-continuous on $\left[0,T\right]$ and $x_{i}\left(nT\right)=x_{i}\left(nT^{-}\right)$. We can solve the optimal level the pulse intensity v when the sequences of impulse point nT are fixed. Let $x\left(t\right)=x^{*}\left(t\right)$, the optimal controls are as follows:

(64) $$\alpha_{2}^{*}\left(t\right)=\left\{\begin{array}{cc} 1, & \frac{\left(\lambda_{2}\left(t\right)-\lambda_{3}\left(t\right)\right)I^{*}\left(t\right)}{A_{3}}\;\geq 1\\ \frac{\left(\lambda_{2}\left(t\right)-\lambda_{3}\left(t\right)\right)I^{*}\left(t\right)}{A_{3}}, & 0<\frac{\left(\lambda_{2}\left(t\right)-\lambda_{3}\left(t\right)\right)I^{*}\left(t\right)}{A_{3}}<1\\ 0, & \frac{\left(\lambda_{2}\left(t\right)-\lambda_{3}\left(t\right)\right)I^{*}\left(t\right)}{A_{3}}\;\leq 0 \end{array}\right.$$

and

(65) $$v^{*}\left(nT\right)=\left\{\begin{array}{cc} 1, & \frac{\left(\lambda_{2}\left(nT^{+}\right)-\lambda_{1}\left(nT^{+}\right)\right)I^{*}\left(t\right)}{B}\geq 1\\ \frac{\left(\lambda_{2}\left(nT^{+}\right)-\lambda_{1}\left(nT^{+}\right)\right)I^{*}\left(t\right)}{B}, & 0<\frac{\left(\lambda_{2}\left(nT^{+}\right)-\lambda_{1}\left(nT^{+}\right)\right)I^{*}\left(t\right)}{B}<1\\ 0, & \frac{\left(\lambda_{2}\left(nT^{+}\right)-\lambda_{1}\left(nT^{+}\right)\right)I^{*}\left(t\right)}{B}\leq 0. \end{array}\right.$$

The optimal control functions can also be simplified as:

(66) $$\alpha_{2}^{*}\left(t\right)=min\left(max\left(0,\frac{\left(\lambda_{2}\left(t\right)-\lambda_{3}\left(t\right)\right)I^{*}\left(t\right)}{A_{3}}\right),1\right),$$

and

(67) $$v^{*}\left(nT\right)=min\left(max\left(0,\frac{\left(\lambda_{2}\left(nT^{+}\right)-\lambda_{1}\left(nT^{+}\right)\right)I^{*}\left(t\right)}{B}\right),1\right).$$

## 6. Simulation

The purpose of this section is to further verify the correctness and practicability of our theory by numerical simulation. We compared the relationship between the pulse charging model, the continuous charging model and the non-charging model. Thus, the advantage of pulse charging over the other two models is received. In Section 6.1, we use the Runge–Kutta [42] method to analyze the stability of pulse charging in MATLAB and compare it with the other two models. The effects of various control parameters on the basic reproduction number of three charging models are analyzed in Section 6.1.

### 6.1. Stable Analysis When $R_{0}<1$

This subsection verifies the stability of the basic reproduction number of the three charging models $R_{i}<1\;(i\;=\;0,1,2)$, where, $R_{0}$ is the basic reproduction number of the pulse charging model, $R_{1}$ is the basic reproduction number of the continuous charging model and $R_{2}$ is the basic reproduction number of the non-charging model. Parameters are set as Λ=0.2, μ=0.004, $\beta_{1}=0.005$, $\beta_{2}=0.005$, $\beta_{3}=0.008$, $\alpha_{1}=0.0001$, $\alpha_{2}=0.001$, γ=0.05, a=0.005, T=10. It is assumed that $N\left(t\right)=S\left(t\right)+I\left(t\right)+A\left(t\right)+LS\left(t\right)+LI\left(t\right)\leq 50$, $S_{0}=48,\;I_{0}=2,\;A_{0}=0,\;LS_{0}=0$ and $LI_{0}=0$.

**Figure 1:**
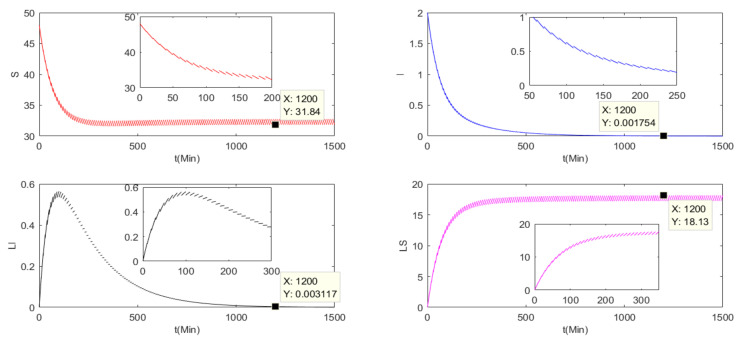
The nodes’ numbers under the pulse charging model with $R_{0}<1$.

Because $R_{0}≜r(P_{2}\Phi_{F-V}\left(t\right))$, we can obtain *R*_0_ = 0.4828 < 1. Therefore, there will be a malware-free *T*-period solution ($\tilde{S}$,0,0,0,$\tilde{LS}$). In Figure 1, all nodes will approach stability. When *T* is approaching infinity, the *I* and *LI* nodes are almost zero, while the *S* and *LS* nodes will be stable at non-zero values. The basic reproduction number of the other two models are calculated by:

(68) $$\begin{array}{c} R_{1}=\frac{\alpha_{1}\Lambda {\left(\gamma +\mu \right)}^{2}}{\left[\left(\beta_{1}+\mu \right)\left(\gamma +\mu \right)-\gamma \beta_{1}\right]\left[\left(\alpha_{2}+\beta_{3}+\mu +a\right)\left(\gamma +\mu \right)-\gamma \beta_{3}\right]},\\ R_{2}=\frac{\alpha_{1}\Lambda }{\left(\alpha_{2}+\beta_{3}+\mu +a\right)\left(\beta_{1}+\mu \right)}. \end{array}$$

**Figure 2:**
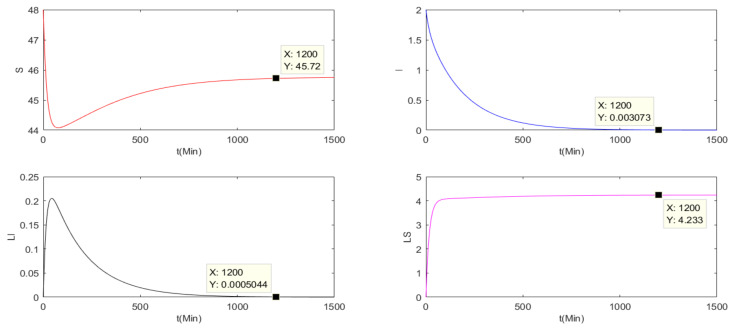
The nodes’ numbers under the continuous charging model with $R_{1}<1$.

**Figure 3:**
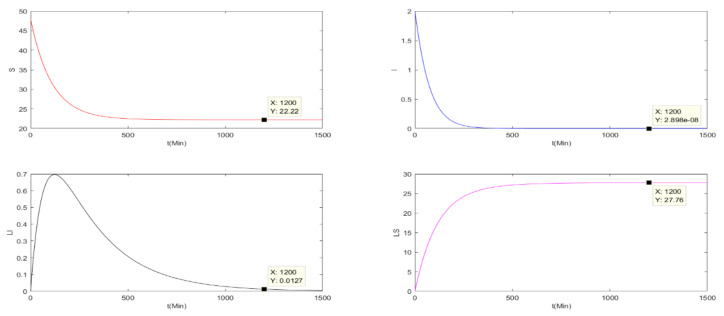
The nodes’ numbers under the non-charging model with $R_{2}<1$.

It is easily obtained that $R_{1}=0.4320<1,R_{2}=0.1481<1$. Thus, the two models both have a global stable malware-free equilibrium point $\left(S\left(t\right),0,0,0,LS\left(t\right)\right)$. As can be seen in Figure 2 and Figure 3, for the continuous charging model and the non-charging model, when t=1200, the number of S are 45.72 and 22.22, respectively. Compared with the pulse charging model in Figure 1, S = 31.84 when t = 1200. It can clearly be seen that the number of S under the pulse charging model is lower than that under the continuous charging model but is higher than that of the non-charged model. However, the relationship among the number of LS is opposite to that among the number of S. Therefore, the pulse charge can better improve the utilization rate of energy and the work efficiency of WRSNs.

### 6.2. Influence of Parameters on the Basic Reproduction Number

Parameters are set as μ=0.004, $\beta_{1}=0.005$, $\beta_{3}=0.008$, $\alpha_{1}=0.0001$, $\alpha_{2}=0.001$, γ=0.05, a=0.005, T=10, $\Lambda \in \;\left[0,\;0.4\right]$, $\beta_{2}\in \left[0,\;0.05\right]$.

**Figure 4:**
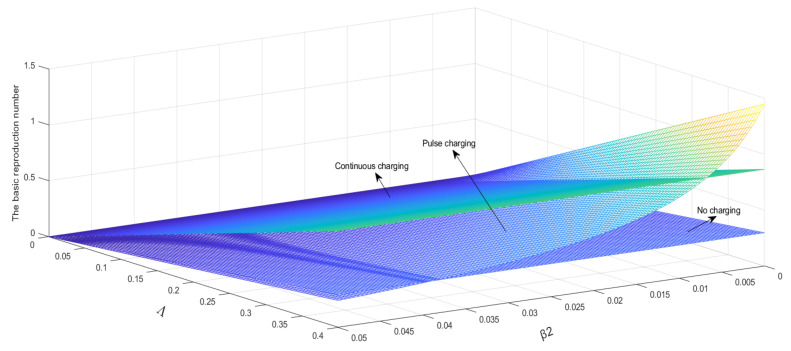
Λ and $\beta_{2}$ relationships with the basic reproduction number.

As shown in Figure 4, the basic reproduction number will increase with the increase in Λ. It is obvious that *β*_2_ under the pulse charging model has greater influence than that under the other two models on the basic reproduction number. It can be seen that some values of the basic reproduction number under the pulse charging model are located between those under the other two models.

**Figure 5:**
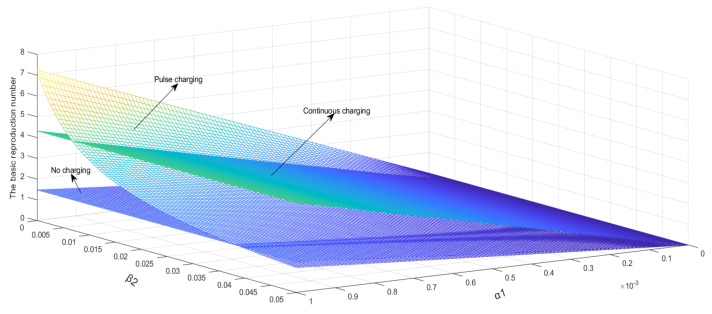
$\alpha_{1}$ and $\beta_{2}$ relationships with the basic reproduction number.

A set of parameters is set as Λ=0.2, μ=0.004, $\beta_{1}=0.005$, $\beta_{3}=0.008$, $\alpha_{2}$ =0.001, γ=0.05, a=0.005, T=10, $\beta_{2}\in \left[0,\;0.05\right]$, $\alpha_{1}\in \;\left[0,\;1\right]$. As shown in Figure 5, it is obvious that the increase in $\alpha_{1}$ increases the basic reproduction number more significantly than that of Λ, and $\beta_{2}$ the influence on the basic reproduction number is almost the same as the change in Figure 4. Thereby determining that $\beta_{2}$ only has a significant impact on the pulsed charging model.

**Figure 6:**
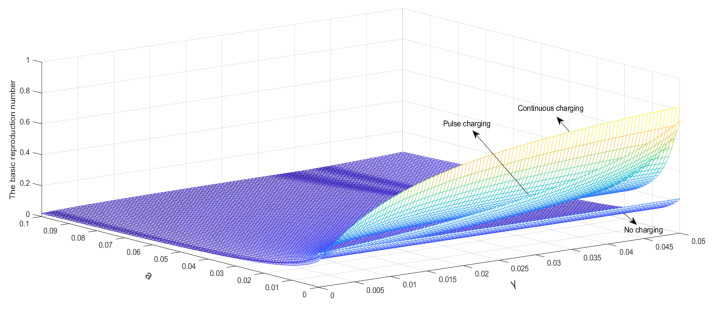
a and γ relationships with the basic reproduction number.

A set of parameters is set as Λ=0.2, μ=0.004, $\beta_{1}=0.005$, $\beta_{2}=0.005$, $\beta_{3}=0.008$, $\alpha_{1}=0.0001$, $\alpha_{2}=0.001$, T=10, $\gamma \in \left[0,\;0.05\right]$, $a\in \;\left[0,\;0.1\right]$. As shown in Figure 6, the basic reproduction number will decrease with the increase in a, and the basic reproduction number will increase with the increase in γ. It is obvious that a has greater influence than γ on the basic reproduction number.

### 6.3. Sensitivity Analysis

In this section, we carefully study the sensitivity of the SIALILS model threshold by evaluating PRCC (partial correlation coefficient). Evenly distributed sampling is carried out for each input parameter of the model, where the maximum value of each parameter is 120% of the sampling baseline value, and the minimum value is 80% of the baseline value. As we know, when the absolute value of PRCC is less than 0.2, the correlation between input parameters and output variables is not significant [43], the absolute value is moderately correlated between 0.2 and 0.4 and highly correlated when the absolute value is greater than 0.4. The distribution interval of each parameter and its corresponding PRCC are shown in Table 3.

**Figure 7:**
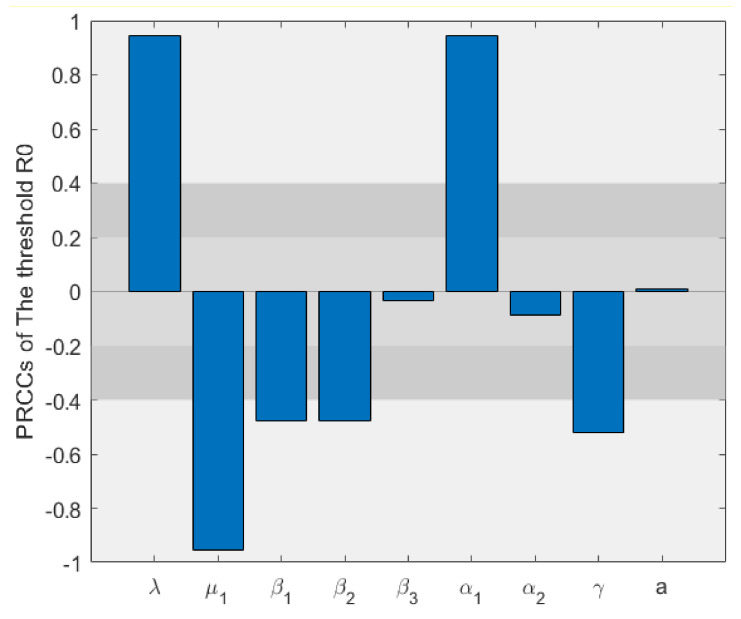
PRCCs of $R_{0}$.

**Figure 8:**
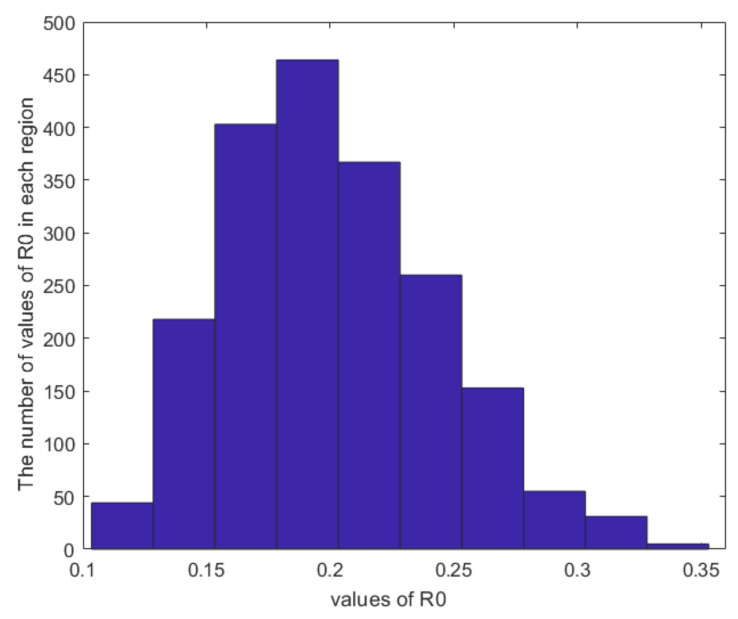
The distribution of the values of $R_{0}$.

From Figure 7, it is obvious that the birth rate λ and metastasis rate of $\alpha_{1}$ are related to the threshold $R_{0}$. However, death rate μ, metastasis rate $\beta_{1}$, $\beta_{2}$ and γ are correlated with threshold $R_{0}$ is significantly negatively correlated, and the remaining parameters are not correlated. This means that if we want to lower the threshold $R_{0}$, we can lower the birth rate λ and metastasis rate $\alpha_{1}$, or increase the death rate *μ*, transfer rate $\beta_{1}$, $\beta_{2}$ and γ. Conversely, we can increase the threshold $R_{0}$ by increasing the birth rate λ and metastasis rate $\alpha_{1}$, or decreasing mortality rate μ, metastasis rate $\beta_{1}$, $\beta_{2}$ and γ. From Figure 8, it can be easily learned that within the range of parameters specified by us, the threshold $R_{0}$ is only between 0.1 and 0.35, and most of them are between 0.15 and 0.25. It can be seen that within this range, the threshold $R_{0}$ have limited changes.

### 6.4. Optimal Control Strategy

In this section, considering the treatment cost of infected nodes, control factors are designed. Four control strategies (MAX control, MIN control, average control, optimal control) are applied to the pulse charging model, and the optimal control problem is numerically simulated. The superiority and effectiveness of the optimal control strategy are verified by comparing the number of nodes in each state and the control cost. Parameters of the four control policies are set as shown in Table 4. The parameters and weight parameters settings of the proposed model are shown in Table 5, where $A_{1},\;A_{2},\;A_{3}\;\mathrm{and}\;B$ are the weight parameters.

**Figure 9:**
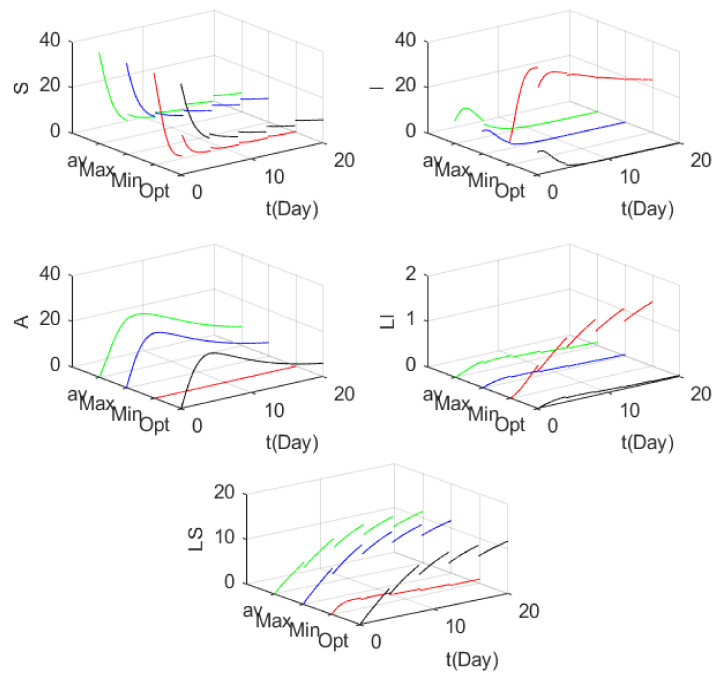
The number of nodes changes with time under the four control strategies.

We set the initial node number as $S_{0}=40,\;I_{0}=10,\;A_{0}=0,\;LS_{0}=0$ and $LI_{0}=0$. The control time is set as 20 days. In Figure 9, the change of the number of nodes of each control strategy over time is shown. Under the MAX control, $v\left(nT\right)=1$*,* the number of I nodes will change greatly at the pulse time to reduce the number of I nodes and increase the number of S nodes so that the model can operate efficiently. In MIN control,$\;v\left(nT\right)=0$, the number of I nodes will greatly increase at the pulse time, and the number of LI nodes will also increase accordingly. Under this control, the number of A nodes is always 0, resulting in the failure of anti-malware A to run normally; the number of S and LS nodes will decrease rapidly. Obviously, MIN control strategy finds it difficult to suppress the spread of malware, leading to the normal operation of anti-malware A, and the number of S and LS nodes will decrease rapidly. Obviously, the MIN control strategy finds it difficult to suppress the spread of malware. On the whole, the number of the S node decreases most obviously under the minimum control strategy, while the number of the I node is completely opposite. The rising rate of the number of the I node under the MIN control strategy is far higher than that of the other three control strategies. It can determine that the MIN control strategy control effect is poorer and the spread of the virus is difficult to control. In the case of the four control strategies, regardless of cost, the Max control strategy effect is the best because it can maintain the high-energy operation of the S nodes and rapidly reduce the number of I nodes to make the system run efficiently. The control effect of the optimal control strategy follows closely.

**Figure 10:**
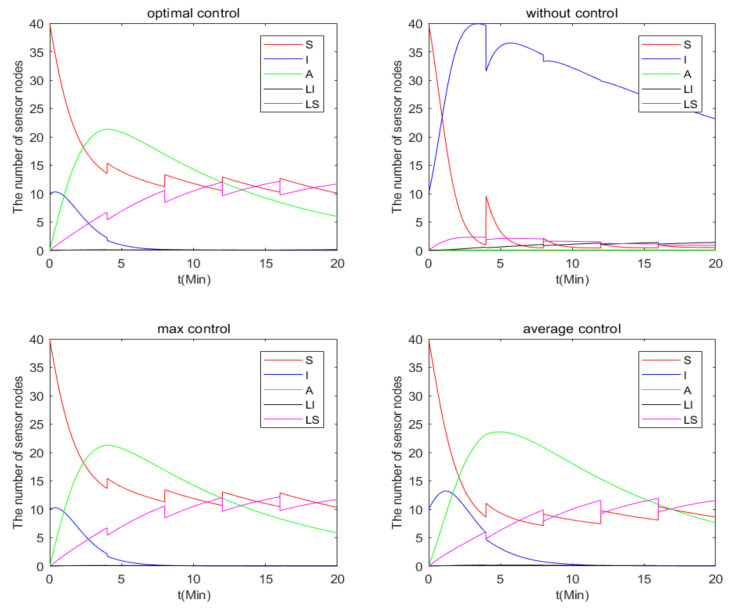
The number of nodes of the four control strategies.

Then, the number of nodes in the model under the four control strategies is further analyzed. According to Figure 10, it can be seen that the number of I nodes rapidly decreases to 0 in optimal control, *MAX* control and average control, and the number of LI nodes hardly changes. However, under *MIN* control, the number of I nodes occupy the majority, and the number of LI nodes and increases accordingly. It is surprising that the number of nodes under optimal control is almost the same as that under *MAX* control, which further verifies the superiority of the optimal control strategy.

**Figure 11:**
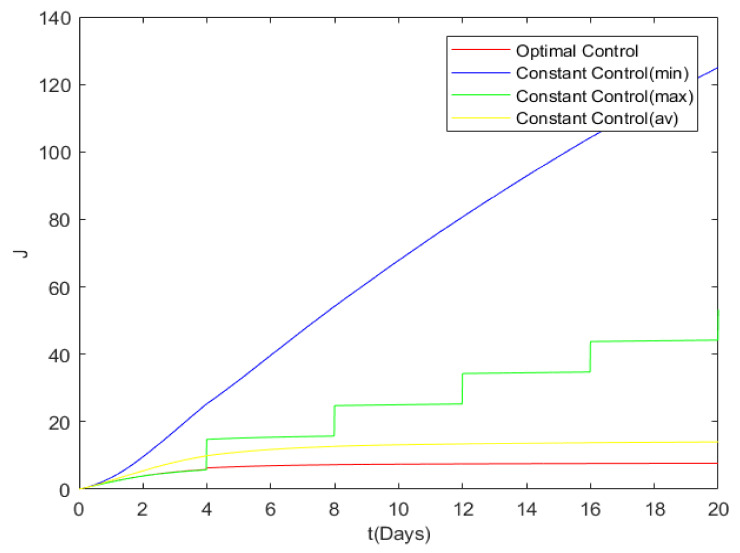
Cost comparison of the four control strategies.

Finally, the cost of the four control strategies is numerically simulated. It is obvious from Figure 11 that *MIN* control has the highest cost, while the optimal control has the lowest cost. The cost of the four control strategies is compared as follows:${\;\mathrm{Cos}t}_{MIN}>{\;\mathrm{Cos}t}_{MAX}>{\;\mathrm{Cos}t}_{AVERAGE}>{\;\mathrm{Cos}t}_{OPTIMAL}.$ The effectiveness of the optimal control is verified.

## 7. Conclusions

In this paper, a novel model of the epidemic based on pulse charging (SIALS-P) for WRSNs is proposed. In each periodic pulse point, the low energy states (LS nodes, LI nodes) are converted into the normal energy states (S nodes, I nodes). The stability of the model is analyzed, and the local and global stability of the malware-free *T*-period solution is also proven. Additionally, the comparison theorem is used to prove the persistence of malware transmission. In order to reduce the model control cost, we propose an optimal control strategy for the proposed model based on the Pontryagin maximum principle. Finally, in the numerical simulation part, we use the Runge–Kutta method to further verify the correctness of the theory and compare the model with the non-charging and continuous charging models. The simulation results show that the number of nodes in pulse charging mode is between those in the other two modes. This suggests that the pulse charging model is more energy-saving when compared with the continuous charging model and has higher working efficiency compared with the non-charging model. The influence of each parameter on the basic reproduction number is given by simulation. PRCC is used to analyze the sensitivity of threshold parameter $R_{0}$ and the feasibility and superiority of optimal control are further verified.

Of course, the study in this paper also has limitations. For example, the simulation results of its periodic or chaotic solutions are unknown. In addition, if a node has an incubation period after infection, it can lead to more complex system behavior (for example, repeated outbreaks, etc.).

## Figures and Tables

**Table 1:** Recent related studies on stability analysis of epidemic models in wireless sensor networks (WSNs).

Authors	Model	Goal
J.D. Hernández Guillén et al. [13]	Susceptible–Carrier–Infectious–Recovered–Susceptible (SCIRS)	Exploring local and global stability of malware-free and epidemic points by analyzing carrier state.
D.W. Huang et al. [14]	Susceptible–Infected–Patched–Susceptible (SIPS)	Through the mechanism of patch injection, analyze local and global stability of epidemic point.
G.Y. Liu et al. [15,16,17,18,19,20,21,22]	Low–Energy–Node (SILS, SILS-P, SISL, SIRS-L, SIALS, ΛSILRD, SILRD, SI1I2L) models	Through the introduction of low-energy nodes, analyze local and global stability of malware-free and epidemic points
S.G. Shen et al. [23]	Vulnerable–Compromised–Quarantined–Patched–Scrapped (VCQPS)	By analyzing the heterogeneity and mobility of sensor nodes in the model, the local and global stability of malware-free points is explored.
R.P. Ojha et al. [24]	Susceptible–Exposed–Infectious– Quarantine–Recovered–Vaccinated (SEIQRV)	By introducing analytical quarantine and inoculation technology, analyze local and global stability of worm-free points.
S. Hosseini et al. [25]	Susceptible–Exposed–Infected–Recovered–Susceptible with Quarantine and Vaccination (SEIRS-QV)	Through the diversification of nodes configuration, analyze local and global stability of malware-free points.

**Table 2:** Research on application of pulse effect in epidemic models.

Authors	Model	Application Area	Goal
A. d’Onofrio et al. [26]	Susceptible–Exposed–Infected–Recovered (SEIR)	Anthroponosis	Pulse inoculation and pulse birth were introduced to analyze the malware-free periodic solution and stability of the malware-free periodic solution. Finally, to prove that PVS (pulse vaccination strategy) is more effective than other vaccination strategies.
D. Zhang et al. [27]	Susceptible–Infected–Removed (SIR)	Anthroponosis	Through the impulsive comparison theorem and analysis technique, prove the existence and stability of the malware-free periodic solution.
Airen Zhou et al. [28]	Susceptible–Infected–Removed (SIR)	Anthroponosis	According to impulsive vaccination occurring at different moments, prove the existence and stability of malware-free periodic solution by using a stroboscopic map.
D. Yu et al. [29]	Susceptible–Infected–Vaccinated–Susceptible (SIVS)	Anthroponosis	Using the impulsive comparison theorem and stroboscopic map, prove the existence and stability of malware-free periodic solution and permanence of the disease.
S.Z. Wang et al. [30]	Susceptible–Infected––Quarantined–Removed–Susceptible (SIQRS)	Anthroponosis	Considering the periodic inoculation of the susceptible population, the stability of the malware-free periodic solution and the persistence of the disease were analyzed using the impulsive comparison theorem.
Z. Zhong et al. [31]	Susceptible–Infected–Removed–Susceptible (SIRS)	Zoonosis	According to birth pulse and impulsive vaccination occurring at different moments, prove the existence and stability of malware-free periodic solution by using the Poincaré map. Through means of the bifurcation theory, discuss the existence of nontrivial periodic solution bifurcated.
X.M. Wang et al. [32]	Susceptible–Infected–Removed (SIR)	Mobile wireless sensor networks (MWSNs)	Based on the pulse differential equation, immune operation is achieved on the susceptible nodes in pulse mode. Additionally, prove the existence and stability of malware-free periodic solutions and obtain the maximum immunization period of time.

**Table 3:** PRCCs values.

Parameters	Range	PRCC
λ	$U\;\left(0.32,\;0.48\right)$	0.9515
μ	$U\;\left(0.24,\;0.36\right)$	−0.9583
$\beta_{1}$	$U\;\left(0.032,\;0.048\right)$	−0.5192
$\beta_{2}$	$U\;\left(0.032,\;0.048\right)$	−0.5406
$\beta_{3}$	$U\;\left(0.0004,\;0.0005\right)$	0.0136
$\alpha_{1}$	$U\;\left(0.0032,\;0.0048\right)$	0.9524
$\alpha_{2}$	$U\;\left(0.008,\;0.012\right)$	−0.1737
γ	$U\;\left(0.12,\;0.18\right)$	−0.5593
a	$U\;\left(0.004,\;0.006\right)$	0.0263

**Table 4:** Parameters setting of four control strategies.

Case	Control Strategy	$\alpha_{2}$	v	J
1	Optimal control	$\alpha_{2}^{*}\left(t\right)$	$v^{*}\left(nT\right)$	$\int_{0}^{\delta T}(A_{1}I\left(t\right)+A_{2}LI\left(t\right)+\frac{A_{3}}{2}\alpha_{2}^{2}\left(t\right))dt+\overset{\eta }{\underset{i=1}{\sum }}\left(\frac{B}{2}V^{2}\left(nT\right)\right)$
2	MIN control	0	0	
3	MAX control	1	1	
4	AVERAGE = control	$\alpha_{2}^{*}\left(t\right)=\frac{\sum_{0}^{T}\alpha_{2}^{*}\left(t\right)}{T}=0.5720$	$v^{*}\left(nT\right)=\frac{\sum_{1}^{\delta }v^{*}\left(nT\right)}{\delta }=0.0587$	

**Table 5:** Parameters setting.

Notation	Value	Notation	Value
λ	0.4	a	0.005
μ	0.04	T	4
$\beta_{1}$	0.05	$A_{1}$	0.6
$\beta_{2}$	0.05	$A_{2}$	0.01
$\beta_{3}$	0.005	$A_{3}$	0.05
$\alpha_{1}$	0.03	B	0.05
γ	0.2		

## Data Availability

Not applicable.
